# Prevalence of Antenatal Anxiety in European Women: A Literature Review

**DOI:** 10.3390/ijerph20021098

**Published:** 2023-01-08

**Authors:** Alba Val, M. Carmen Míguez

**Affiliations:** Department of Clinical Psychology and Psychobiology, Faculty of Psychology, University of Santiago de Compostela, Campus Vida, 15782 Santiago de Compostela, Spain

**Keywords:** epidemiology, prevalence, pregnancy, prenatal, antenatal, anxiety, generalized anxiety disorder, Europe

## Abstract

The presence of anxiety during pregnancy is associated with adverse consequences for both mothers and their babies. The aim of this study was to review the prevalence of anxiety in European pregnant women in order to find out which countries have published the most studies in respect to the presence of anxiety during pregnancy, which countries are the most and least prevalent in terms of anxiety within pregnant women, and which are the most common tools used to assess anxiety during this stage. As such, a literature review was conducted regarding the studies that were published in the last twenty years in the PsycInfo, Medline, and SCOPUS databases. Thirty-eight studies were selected for the purposes of this review. The prevalence of anxiety in pregnancy and generalized anxiety disorder (GAD) varies considerably between studies. The European countries that have carried out the most research on this issue are Spain, Italy, and the United Kingdom. The most widely used assessment instrument is the State Trait Anxiety Inventory (STAI). The lowest prevalence rate of anxiety, using the STAI-E, was found in Poland, 7.7%, and the highest was found in Italy, 36.5%. The prevalence of GAD ranges from 0.3% to 10.8%. This indicates that anxiety in pregnant women is a very relevant mental health problem. It is therefore important to detect and intervene early in order to promote the well-being of both mothers and children.

## 1. Introduction

Pregnancy was traditionally considered a time of happiness for women [1]; further, it was believed to be a protective factor with respect to the onset of mental disorders. However, it has now been shown that not only does it not protect women but it is a period of vulnerability in which pregnant women may develop a mental disorder [2,3]. Specifically, it is estimated that 20% of women may develop a mental disorder during the perinatal stage, mainly anxiety and depression [4,5]. It must be noted that, when compared to the pregnancy period, the postnatal period has been investigated more frequently. One reason for this may be the belief that women are “hormonally protected” from mental disorders during pregnancy [6]. Additionally, women themselves may be reluctant to share symptoms of sadness or anxiety during gestation, as socially it should be a time of happiness [1,7]. In addition, at the professional level, there is a tendency to focus on physical health (maternal and fetal) during pregnancy, rather than mental health. In addition, there is a tendency to attribute emotional complaints to the physical and hormonal changes that occur during gestation [8]. Added to this, many pregnant women present themselves with somatic symptoms and complaints that are characteristic of anxiety or depression, such as fatigue, loss of energy, or sleep disturbances [9]. Therefore, due to this fact, it can be difficult to distinguish between common symptoms of pregnancy and symptoms of depression or anxiety [10,11].

In recent years, there has been an increased interest in the prenatal stage, which has led to the knowledge that around 20–29% of pregnant women present a psychopathological disorder [12,13,14,15,16].

As such, anxiety during pregnancy is a very common disorder [17], and certain studies have suggested that it is higher in pregnant women than in the general population. However, the data must be interpreted with caution due to the fact that, currently, results are inconclusive. For instance, Adewuya et al. [18] found that 39% of pregnant women met the criteria for a diagnosis of an anxiety disorder—including panic disorders, specific phobias, social phobias, obsessive compulsive disorder, generalized anxiety disorder, posttraumatic stress disorder, and anxiety disorders associated with medical conditions—when compared with 16.3% of non-pregnant women. Likewise, Viswasam et al. [19], in a review and meta-analysis on the prevalence and onset of anxiety disorders during pregnancy, found that the prevalence of panic disorders and obsessive–compulsive disorders was higher in pregnant women (3%) than in the general population (1.6%). Other authors [16,20] have reported that, although the prevalence rate of anxious symptomatology and anxiety disorders was high among pregnant women, the evidence on whether this differed from the prevalence in the general population was inconclusive.

Different reviews conclude that anxiety is very common in the pregnant population worldwide. For example, Leach et al., in Australia [21], found that between 6.8% and 59.5% of pregnant women experienced anxiety symptoms; in addition, between 2.6% and 39% suffered from some type of anxiety disorder. In Canada, Dennis et al. [22] observed that anxiety symptoms during pregnancy were present in 22.9% of women and that a diagnosis of an anxiety disorder was found in 15.2%. Specifically, GAD was present in 4.1% of pregnant women. Likewise, Fawcett et al., also in Canada [23], found that 20.7% of pregnant women met the criteria for a diagnosis of anxiety, thereby determining that GAD was one of the most prevalent disorders during pregnancy. Recently, in the United Kingdom, Nielsen-Scott et al. [15] indicated that 29.2% of women prenatally reported anxiety and 8.1% possessed an anxiety disorder. This disparity in the prevalence of anxious symptomatology and anxiety disorders may be due to the use of different assessment instruments and cut-off points, as well as to cultural differences in relation to the importance attached to mental health in a given country and/or culture.

The presence of anxiety during pregnancy has been associated with various consequences for mothers, such as an increased likelihood of developing postpartum depression [24], as well as in an increased risk in regard to preeclampsia, obstetric complications, and bonding problems [25,26,27]. As for newborns, it is associated with a lower gestational age, birth weight, and poor cognitive development, among others [28,29]. Therefore, it is important to detect and intervene early with respect to prenatal anxiety in order to promote the well-being of mothers and children.

Although there is increased interest in maternal mental health, there is a specific lack of studies that have been conducted on different continents that focus on anxiety during pregnancy. In fact, only one review [30] was found in Africa that aimed to determine the prevalence of psychological disorders in women during pregnancy, as well as after childbirth. The prevalence of anxiety was found to be present in 14.8% of African pregnant women.

Although we know that anxiety affects a large number of women during pregnancy, no such review has been carried out in Europe to determine which countries have conducted the most research on the subject and which possess a higher prevalence rate. This is a key matter that requires further investigation as cultural and socio-economic differences between certain countries are important to consider. Likewise, knowing the prevalence data in different trimesters will allow us to elucidate how anxiety evolves throughout the pregnancy.

Another relevant aspect is to know what are the most commonly used instruments that are utilized in order to evaluate anxiety, as well as the cut-off points established for each one. This should be performed with the purpose of attempting to determine what tool is most commonly used in Europe in order to measure anxiety during this stage, as well as what is the most adequate cut-off point in order to better identify the pregnant women who suffer from anxiety.

When referring to anxiety in general we will refer to the anxious symptomatology that is assessed via screening instruments. A woman is considered to have high levels of anxiety if she exceeds the cut-off point as recorded by the screening instrument. Generalized anxiety disorder (GAD) refers to the diagnosis of anxiety according to the criteria established by DSM, for example. As such, a woman will be diagnosed with GAD if she meets these criteria.

The main objective of the present review is to update our understanding in respect to the current magnitude of antenatal anxiety in European women. GAD is one of the most common disorders in the general population [31,32], which leads us to assume that it will also be prevalent during pregnancy. This disorder refers to the presence of anxiety in everyday life in general, without being related to any specific type of anxiety. In this regard, it would be the diagnostic category that is closest to the anxious symptomatology assessed by self-report instruments. The specific objectives of this study, thus, are: (a) to know which European countries have published more in respect of the presence of anxiety during pregnancy, which would help us to understand which countries are more concerned regarding the mental health status of pregnant women; (b) where countries are in relation to each other in respect to the highest and lowest prevalence rates; and (c) what instruments are the most commonly used in order to assess anxiety during this stage.

## 2. Method

A review of publications on the prevalence of anxiety during pregnancy in Europe over the last 20 years was carried out utilizing PRISMA guidelines. The databases consulted were PsycINFO, Medline, and SCOPUS. The keywords used for the search were: “prevalence” AND “anxiety” AND “generalized anxiety disorder” AND “pregnancy” OR “prenatal” OR “antenatal”.

The following inclusion criteria were used in order to select the studies: (1) articles published between January 2001 and December 2021; (2) articles published in the English language; (3) articles that included the search keywords in the title and/or abstract; (4) articles that used self-report measures in order to measure anxious symptomatology and/or clinical interviews in order to assess GAD; and (5) articles that provided data from European countries.

**Figure 1:**
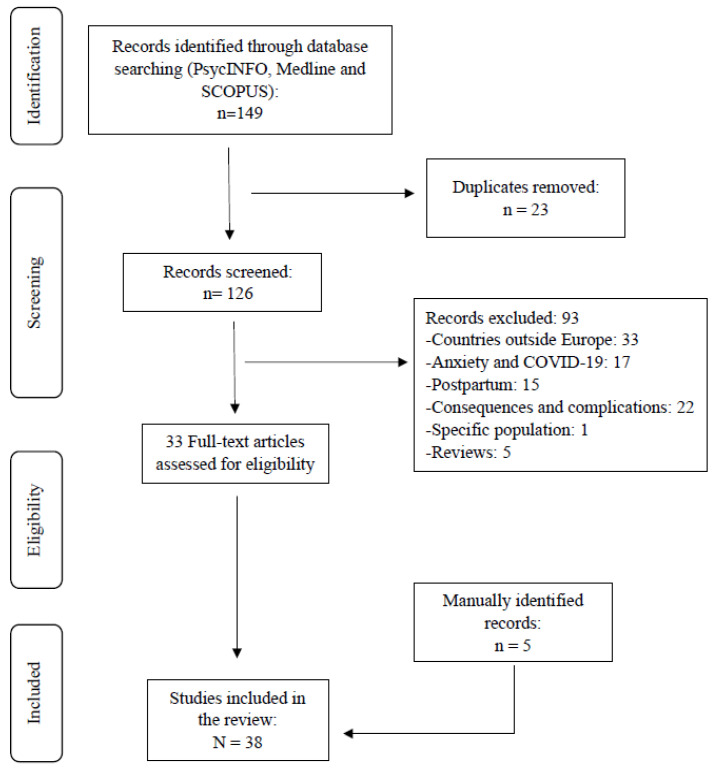
Flowchart of study selection.

The initial search yielded a total of 149 articles. Twenty-three were eliminated due to the fact that they were duplicates. Ninety-three were discarded because of their title and/or abstract due to the fact that: 33 were from countries outside Europe; 17 were on the prevalence of anxiety related to the coronavirus pandemic (COVID-19); 15 reported on postpartum; 22 were related to complications and consequences of anxiety during this stage; 1 pertained to a specific population; and 5 were reviews. Thus, 33 articles were selected for the purposes of full-text reading. In addition, 5 articles obtained via a manual search (articles found from the bibliography of others) were added. Thus, the final sample comprised 38 publications (Figure 1).

### Data Extraction and Quality Assessment

The first author screened each article by title and abstract, thus retrieving the articles that met the inclusion criteria. The second author independently screened one-third of the articles from the electronic search. Any disagreements over study inclusion were resolved through discussion between the two authors. The first author extracted the following data from each article: author names; year of publication; sample size; total prevalence; the number of those assessed; the individual prevalence of each assessed; the structured diagnostic interview used (e.g., MINI, SCID, DIS); the diagnostic criteria used (ICD-10, DSM-IV); the country/region that the study was conducted in; average gestational week; and the medically based exclusion criteria (e.g., severe medical problems in the mother, fetal malformation, pregnancy complications, etc.). The second author checked this information for accuracy. The methodological quality of each study was evaluated on the basis of six criteria: (1) clear study aims; (2) clear inclusion and exclusion criteria; (3) valid measurement of mental health; (4) good response rate; (5) adequate description of data; and (6) appropriate statistical analysis.

## 3. Results

**Figure 2:**
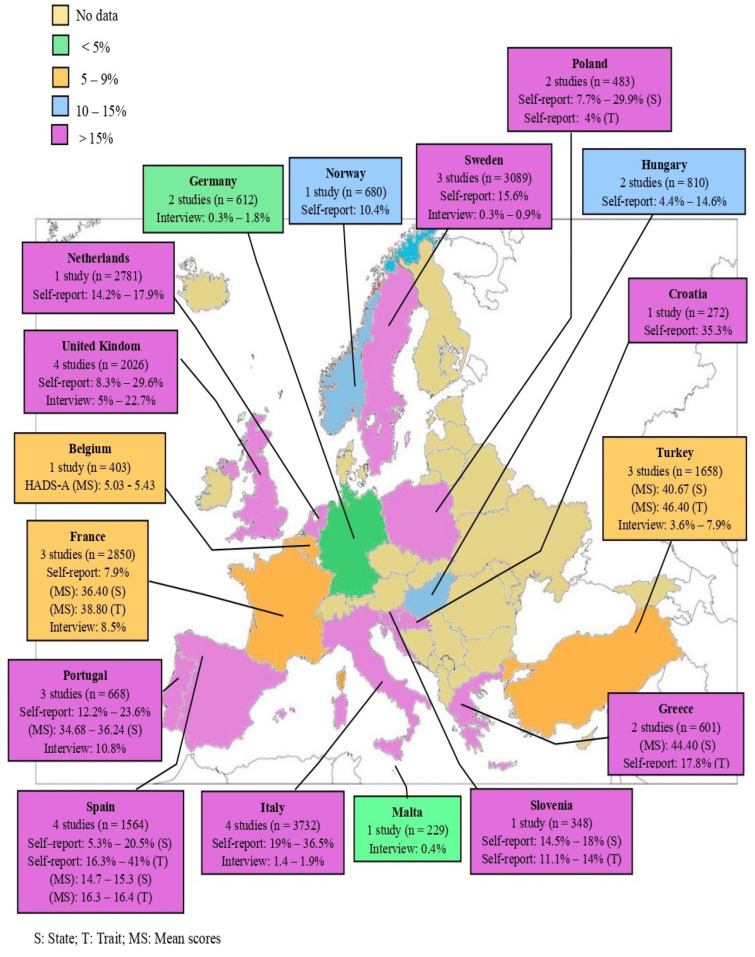
Distribution of prenatal anxiety across European countries.

The present review provides anxiety prevalence data from 17 European countries (i.e., Italy, the United Kingdom, Poland, Spain, Turkey, Croatia, the Netherlands, Portugal, Germany, Slovenia, Sweden, Greece, Hungary, France, Belgium, Malta, and Norway). The studies reviewed vary in the number of subjects in the sample and their characteristics, as well as in the instrument used to assess anxiety during pregnancy and in the prevalence ranges found (Figure 2).

Table 1 shows that the State Trait Anxiety Inventory (STAI) [33] was the most widely used instrument that was utilized in order to estimate the presence of anxiety during the prenatal stage; this was determined by the fact that it was used in 18 of the 38 selected studies. Other self-report instruments used to measure anxiety during this stage were: the Hospital Anxiety and Depression Scale (HADS) [34], the Generalized Anxiety Disorder Anxiety Scale (GAD-7/GAD-2) [35], and the anxiety subscale of the Edinburgh Postnatal Depression Scale (EPDS) [36].

### 3.1. Prevalence of Anxiety with the STAI

The STAI provides two types of anxiety measures: Trait anxiety (STAI-T) and State anxiety (STAI-S). The prevalence of state anxiety during pregnancy varies among the different European countries, with the lowest prevalence at 7.7% (STAI ≥ sten 7) in Poland [40] and the highest at 36.5% (STAI ≥ 40) in Italy [39].

In Italy, Cena et al. [37] employed two tools in order to measure anxiety during pregnancy: the STAI-S (≥40) and the anxiety subscale of the Edinburgh Postnatal Depression Scale, i.e., the EPDS-3A (≥6). They found that 19% of women exceeded the cutoff point of one or both scales.

Certain studies [48,51,62] also provide the evolution of state anxiety in the different trimesters of pregnancy. Canário and Figueiredo [48], in Portugal, obtained mean scores of 36.04, 34.68, and 36.24 for the first, second, and third trimester of gestation, respectively. Likewise, rates of 13.1%, 12.2%, and 18.2% (STAI ≥ 45) were found in the first, second, and third trimester of pregnancy, respectively [62]. In Slovenia, Podvornik et al. [51] found a prevalence of 18% in the first trimester, 15.4% in the second, and 14.5% in the third (STAI ≥ 45). In Spain, Vázquez and Míguez [41] conducted a longitudinal study with a sample of 569 pregnant women and observed that the anxious symptomatology varied throughout pregnancy. The prevalence with STAI (≥32) was 10% in the first trimester, 5.3% in the second trimester, and 7.2% in the third trimester.

Regarding the studies that have focused on assessing anxiety [40,42,51,59,60,70], the highest rate was found in Italy [60], where figures of 25.3% (STAI > 40) were reached, and the lowest rate, 4.0%, was found in Poland in primiparous mothers [40], whereby the STAI-R (sten 8 or higher) scale was utilized.

Regarding mean scores, the highest was found in Turkey [59], 46.40, within low-income women, and the lowest, 37.78, was found in Poland [40].

### 3.2. Prevalence of Anxiety with HADS-A

In addition to the STAI, the next most commonly used self-report instrument, which was utilized specifically in 6 of the 38 selected studies, has been the HADS anxiety subscale. In regard to this instrument, in Poland, it has found anxiety rates of 29.9%, where ≥8 was set as the cut-off point [57]. In contrast, in Norway, Berle et al. (2005) found the lowest rate, i.e., 10.4% (HADS-A > 8) [66].

Regarding the evolution of anxiety, in the Netherlands [47], it was found that the level of anxiety in the first weeks of pregnancy (10–12) and in the weeks closest to delivery (34 weeks), a decrease from 17.9% to 14.2%. In Poland, Makara-Studzinska et al. [57] were also interested in this evolution, and they obtained a percentage of 27.4% anxiety in the first trimester of pregnancy, 23.9% in the second, and 29.9% in the third. The evolution of anxiety had been previously investigated in Belgium by van Bussel et al. [64], and they obtained mean scores of 5.03, 5.00, and 5.43 in the first, second, and third trimester, respectively.

### 3.3. Prevalence of Anxiety with GAD

In order to estimate the prevalence of prenatal anxiety, certain authors have also used the GAD-7 and GAD-2. In Spain, Soto-Balbuena et al. [46] utilized the GAD-7 (≥7) and obtained scores that varied according to the trimesters. In the first trimester, they found that 19.5% of women had anxiety symptoms, with anxiety decreasing to 16.8% in the second trimester and increasing again in the third trimester to 17.2%, although this was conducted without reaching initial levels. In the United Kingdom, Savory et al. [38] found that 8.3% of women in early pregnancy possessed high levels of anxiety (GAD-7 ≥ 10). Likewise, Nath et al. [44], using two items of this scale (GAD-2 ≥ 3), found that 23% of women presented anxiety during pregnancy.

### 3.4. Prevalence of Generalized Anxiety Disorder (GAD)

This review also included studies that evaluated the presence of anxiety as measured by clinical diagnostic interviews, with the aim of determining the presence of GAD in women during their pregnancy. Specifically, in 13 of the reviewed studies clinical interviews were used. The structured clinical interview for DSM-IV-TR, (SCID) [72], has been the most widely used for the purposes of detecting anxiety disorders (this was the case in 7 of the 13 reviewed studies). The prevalence rates found for GAD were lower than those found by self-reports, but they also demonstrated great variability, with rates ranging from 0.3% in Germany, Sweden, and Malta [14,58,68] to 22.7% in the United Kingdom [63].

Likewise, a study in Germany [58] shows the evolution of GAD throughout the different trimesters of pregnancy, thereby obtaining a rate of 1.3% in the first, 0.3% in the second, and 1.8% in the third trimester.

### 3.5. Evolution of Anxiety during Pregnancy

It is difficult to establish the pattern of anxiety throughout pregnancy, as the data varies according to the instrument used and the country. Moreover, few studies assess anxiety in the different trimesters of pregnancy.

While utilizing the STAI-E, research conducted in Portugal [48,62] found that anxiety followed a “V”-shaped pattern—i.e., anxiety was found to be higher in early and late pregnancy and then decreased in the second trimester. Specifically, Canário and Figueiredo [48] observed that the highest levels of anxiety occurred in the first trimester, whereas Figueiredo and Conde [62] observed that the time of highest anxiety was in the third trimester. In Spain, Vázquez, and Míguez [41] also found a similar trajectory; they detected the highest levels of anxiety during the first trimester. Podvornik et al. [51], in Slovenia, observed that anxiety progressively decreased from the beginning of gestation to its end. In Italy, Cena et al. [39]—when evaluating the second and third trimester of pregnancy—found that the highest level of anxiety occurred in the second trimester.

The data obtained with HADS-A also showed great variability. In Poland, it was found to also follow a V-shaped trajectory [57], as was the case in Portugal and Spain. In Belgium, van Bussel et al. [64] found that anxiety was stable in the first and second trimester and then slightly increased in the third trimester (_/), thereby observing a significant increase in the mean total HADS-A in the third trimester of pregnancy when compared to the first and second trimesters. In the Netherlands, van de Loo et al. [47], when assessing early and late pregnancy, observed that anxiety was higher in the first weeks of gestation than in the last few weeks.

Employing the GAD-7 (≥7), in Spain, Soto-Balbuena et al. [46] found that 19.5% of women possessed anxiety symptoms in the first trimester, which then declined to 16.8% in the second trimester and then increased again in the third (17.2%). Again, they also found a V-shaped evolution in terms of trajectory.

Despite the diversity of data found in the self-report measures on the evolution of anxiety during pregnancy, the pattern that is most repeated with different instruments is a “V”-shaped evolution of anxiety. That is to say that the presence of greater anxious symptomatology at the beginning of pregnancy decreases as the pregnancy progresses and then increases again in the weeks prior to delivery. Of the six studies that found this pattern, three [41,46,48] indicated that the highest levels of anxiety are reached during the first trimester and the other three [57,62,64] in the third trimester. This latter pattern was also found by Martini et al. [58], in Germany, while using the diagnostic interview in order to establish the evolution of GAD throughout the trimesters of pregnancy, whereby they obtained a higher prevalence during the last trimester. However, these data should be interpreted with caution due to the fact that the authors did not indicate that the differences in anxiety between trimesters were significant [41,46,48,57,62] or that they did not find any differences between trimesters [51].

## 4. Discussion

One of the aims of this review was to determine which European countries have published the most regarding the presence of anxiety during pregnancy. It was found that, in Europe, the countries that—over the last 20 years—have conducted the most research on the subject are Spain, Italy, and the United Kingdom, with four studies each. They are then followed by France, Portugal, Turkey, and Sweden with three studies. Poland, Greece, Germany, and Hungary contributed two each. Further, the other countries that have carried out one study on the presence of anxiety during pregnancy are Croatia, the Netherlands, Belgium, Malta, Norway, and Slovenia. No studies were found from Austria, Bulgaria, Cyprus, Czech Republic, Denmark, Estonia, Finland, Ireland, Latvia, Lithuania, Luxembourg, and Romania.

Regarding the prevalence rates of anxious symptomatology and GAD during pregnancy in Europe, the results found show that the prevalence of anxiety, when using self-report instruments, ranges from 7.7% (STAI ≥ sten 7) to 36.5% (STAI ≥ 40). The prevalence of GAD, when using a clinical interview, ranges from 0.3% to 22.7%. A fact that is of note, the highest rate of GAD, 22.7%, was obtained in a study that included women with high scores in regard to screening measures [63]. If we eliminate this data, the prevalence of GAD would range from 0.3% to 10.8%. Despite the instances of very high data found, it is likely that the prevalence provided does not reflect the true magnitude of such a problem, as emotional distress during pregnancy tends to be underestimated due to the stigma that not feeling well during this stage of life can bring. In addition, most studies measure anxiety at a single point in time; as such, they do not take into account the evolution of anxiety throughout the different trimesters of pregnancy. This is significant due to the fact that, as with depression [73], we know that it can vary throughout the stages of pregnancy.

The occurrence of anxiety during pregnancy is high worldwide, although the prevalence rates may be modified according to culture. The European continent is composed of countries with different cultures and different socioeconomic statuses, which may help—to a certain degree—in explaining the large variability in the prevalence rates found.

Regarding clinical interview data, data from Canada show that 15.8% of pregnant women meet the criteria for an anxiety disorder (according to the criteria detailed in the DSM-IV-TR [74]). Furthermore, Buist, Gotman, and Yonkers [75] found that 9.5% of pregnant women in Australia met the criteria for generalized anxiety disorder. Moreover, in southeast Africa, it was found that 23% of pregnant women met the criteria for one of the anxiety disorders that are included in the DSM-IV-TR [76]. The data from this review indicate that, in Europe, the proportion of pregnant women with a diagnosis of GAD is lower than the data found in other countries of the world, such as Canada and Southeast Africa, but includes data that are similar to those found in Australia.

Regarding the prevalence found by self-report measures, the European countries where the greatest similarity is found are in Italy, Croatia, Poland, and the United Kingdom—i.e., countries in which higher rates of prenatal anxiety are found (29.6–36.5%). This is a matter that can be explained by the fact that these studies utilized the same measurement instrument and a similar cut-off point (STAI ≥ 40). The lowest rates of anxious symptomatology were obtained by Poland, France, and Norway at 7.7%, 7.9%, and 10.4%, respectively. In the case of France and Norway, this may be due to the fact that these countries allocate more resources to addressing mental health problems, and there are thus a greater number of professionals working in this field [77].

Regarding the prevalence of the GAD diagnosis, we can observe that Malta (0.3%), Sweden (0.3–0.9%), Germany (0.3–1.8%), and Italy (1.4–1.9%) possess the lowest rates, while the United Kingdom possesses the highest (5.0–22.7%). The discrepancy between the data provided in Italy, according to the instrument used to assess anxiety (i.e., self-report vs. clinical interview), is striking. Indeed, this corroborates with the fact that self-reports provide higher prevalence rates.

This disparity in the data can be explained by the absence of a “gold standard” for measuring anxious symptomatology during the prenatal stage and also due to the different cut-off points used, which is a matter that requires agreement. To this, we must add the methodological limitations of certain studies, such as the use of cross-sectional designs—which only provide data from a specific moment in time, not allowing us to analyze the evolution of anxiety throughout the different trimesters—or the use of small samples, which limit the reliability of the data and their generalizability. Cultural differences must also be taken into account, due to the fact that it is known that the way in which anxiety is experienced varies according to different cultures. That is, expectant mothers may overestimate or underestimate their response to questionnaires depending on their beliefs, preconceptions, cultural context, and the degree of mental health stigma in their culture [78]. Understanding the cultural differences in women’s experiences and emotions regarding childbearing should be the starting point for designing effective screening tools or intervention strategies [79].

Regarding the evolution of anxiety throughout the different trimesters of pregnancy, the pattern that is most repeated is a “V”-shaped evolution. That is to say that the presence of greater anxious symptomatology at the beginning of pregnancy decreases as the pregnancy progresses, and then increases again in the weeks prior to delivery. It is possible that, as indicated by Rallis et al. [80], this trajectory of anxiety can be explained by a series of factors that occur during the first weeks of gestation, such as the risk of miscarriage and nausea, as well as in physical, hormonal, and emotional changes—which may increase the vulnerability in respect to developing anxious symptoms. On the other hand, increased anxiety in the third trimester may be due to increased physical discomfort, the proximity of childbirth, and factors associated with the impending life change that is brought about by childbirth.

Another objective we set for ourselves was to determine which instruments were the most commonly used in respect to assessing anxiety during this stage. We found that most studies use the STAI. However, this tool does not assess anxiety that is specific to pregnancy, such that it may not be detecting all the anxiety symptoms that can occur during this stage. Another problem with the STAI is the great variability of cut-off points that have been used in different countries, ranging from ≥32 to ≥45 for state anxiety and from ≥40 to >52 for trait anxiety, which could contribute toward explaining the variability in the prevalence rates of anxiety during pregnancy from one country to another. The next most commonly used instrument to measure anxiety during the prenatal stage was the HADS anxiety subscale (HADS-A), which is composed of seven items measuring general anxiety. However, some of the items used to assess anxiety can be confused with pregnancy-related symptoms, which can inflate the prevalence rate of anxious symptoms by detecting false positives [81]. In fact, in this review, the range of prevalence found by the HADS-A (10.4–29.6%) was higher than that found with the GAD (8.3–23%).

Fewer studies, i.e., 13 of 38, have used the clinical interview in order to diagnose generalized anxiety disorder. It should be taken into account that the use of clinical interviews involves more application time than self-report measures. In addition, it is necessary to ensure that mental health professionals are trained in their application. The most commonly used interview during the prenatal stage was the SCID, an interview that corresponds to DSM criteria. It is thus important to utilize clinical interviews in order to establish more reliable prevalence rates of anxiety. In the case of prenatal and postpartum anxiety, a problem with the DSM-5 criteria diagnosis was found in the requirement for excessive worries to be present for at least 6 months [82]. Based on this criterion, certain pregnant or postpartum women may potentially be excluded. Therefore, there are certain investigators who may wish to consider the presence of GAD during pregnancy if other DSM criteria are met for a minimum duration of one month [75]. As such, it would be important for these interviews to develop specific criteria for this stage of life.

Various limitations in this study should be considered when interpreting the findings obtained. First, the number of subjects in the sample and their characteristics vary from one study to another. For example, certain studies [54,71] utilized a sample size that is not representative of the population. Likewise, other studies evaluated less socially favored samples [59] or take into account parity [40]. This limits the generalizability of the results found. Another limitation is found in the tools that were used to detect anxiety. In most of the investigations, self-reports were utilized instead of clinical interviews, and, in turn, the cut-off points also varied from one country to another. The absence of a gold standard for measuring anxiety during this stage, as well as in the lack of a well-established cut-off point for identifying women with anxiety, may limit the accuracy of establishing prevalence ranges. Finally, the diversity of the research designs that were employed (i.e., cross-sectional vs. longitudinal) also limits interpretations.

## 5. Conclusions

It is clear that anxiety in pregnancy is very frequent among European pregnant women. However, not all countries have dedicated efforts to investigate this problem, which has important implications due to the fact that it has been associated with adverse consequences for mothers and their babies. The lack of data from certain European countries remains to be further investigated. Future research should determine the role of pregnancy on maternal mental health. This would thus require longitudinal studies that assess women before, during, and after pregnancy. 

Awareness of the importance of maternal mental health needs to be raised. Furthermore, women should be routinely evaluated in their follow-up and pregnancy monitoring visits, as this would allow for early detection and treatment before the symptomatology becomes more severe, thereby reducing the negative consequences resulting from anxiety in pregnancy.

## Figures and Tables

**Table 1:** Prevalence of anxiety during pregnancy.

Study	N (Pregnant Women)	Anxiety Measure	Time of Evaluation	Prevalence (%)	
Cena et al. (2021) Italy [37]	934	STAI-S (≥40) EPDS-3A (≥6)	3rd trimester (27–40 weeks)	19.0	
Savory et al. (2021) United Kingdom [38]	302	Self-reported mental health problems GAD-7 (≥10)	Pregnancy (≤18 weeks)	22.2 8.3	
Cena et al. (2020) Italy [39]	1142	STAI-S (≥40)	Pregnancy 2nd trimester 3rd trimester	24.3 36.5 22.8	
Kiepura and Kmita (2020) Poland [40]	169 primiparous	STAI (mean scores) STAI-S (sten ≥ 7) STAI-R (sten ≥ 8)	Pregnancy (24–37 weeks)	State anxiety: 34.73 7.7 Trait anxiety*:* 37.78 4	
Vázquez and Míguez (2020) Spain [41]	569	STAI (≥32)	1st trimester 2nd trimester 3rd trimester	10 5.3 7.2	
González-Mesa et al. (2019) Spain [42]	250 Turkish 264 Spanish	STAI Mild: 0–30 Moderate: 30–44 Severe ≥ 45	Pregnancy (10–12 weeks)	*State anxiety:* Mild: 56.8 Moderate: 14.7 Severe: 20.5 *Trait anxiety:* Mild: 31.4 Moderate: 19.7 Severe: 20.2	
Uguz et al. (2019) Turkey [43]	1154	SCID-I	Pregnancy	7.9	
Nath et al. (2018) United Kingdom [44]	528	SCID (DSM-IV) GAD-2 (≥3)	Pregnancy	5.0 (GAD) 23.0	
Nakić Radoš et al. (2018) Croatia [45]	272	STAI (≥40)	Pregnancy	State anxiety 35.3	
Soto-Balbuena et al. (2018) Spain [46]	385 286 261	GAD-7 (≥7)	Pregnancy: 1st trimester 2nd trimester 3rd trimester	19.5 16.8 17.2	
van de Loo t al. (2018) Netherlands [47]	2781 2167	HADS-A (≥8)	Pregnancy: 10–12 weeks 34 weeks	17.9 14.2	
Canário and Figueiredo (2017) Portugal [48]	260	STAI-S (>40)	Pregnancy 8–14 weeks 20–24 weeks 30–34 weeks	Mean scores 36.04 34.68 36.24	
Dikmen-Yiliz et al. (2017) United Kingdom [49]	950	HADS-A (≥10)	Pregnancy (26–35 weeks)	29.6	
Martini et al. (2015) Germany [50]	306	CIDI-V GAD	Pregnancy 12–17 weeks 22–24 weeks 35–37 weeks	Incident	Recurrent
				0.7	18.2
				0	1.3
				0	0.3
				0.7	1.0
Podvornik et al. (2015) Slovenia [51]	348 100 117 131	STAI (≥45)	Pregnancy: 1st trimester 2nd trimester 3rd trimester	STAI-S	STAI-R
				15.8	12.5
				18.0	11.1
				15.4	12.1
				14.5	14.0
Rubertsson et al. (2014) Sweden [52]	916	HADS-A (≥8)	Pregnancy: (8–12 weeks)	15.6	
Koutra et al. (2014) Greece [53]	438	STAI-T (≥48)	Pregnancy: 3rd Trimester	17.8	
Tendais et al. (2014) Portugal [54]	148	STAI-S (≥45) SCID	Pregnancy (8–34 weeks)	23.6 10.8 (GAD)	
Bödecs et al. (2013) Hungary [55]	503	STAI-T (>52 clinical anxiety) STAI-T (48 a 52 subclinical anxiety)	Pregnancy: (average 8 weeks)	Total: 14.2 Clinical anxiety: 4.4 Subclinical anxiety: 9.8	
Gourounti et al. (2013) Greece [56]	163	STAI-X (mean scores)	Pregnancy: 11–26 weeks	44.4	
Makara-Studzinska et al. (2013) Poland [57]	314	HADS-A (≥ 8)	1st trimester 2nd trimester 3rd trimester	27.4 23.9 29.9	
Martini et al. (2013) Germany [58]	306 293 278	CIDI-V	1st trimester 2nd trimester 3rd trimester	GAD: 1.3 GAD: 0.3 GAD: 1.8	
Kavlak et al. (2013) Turkey [59]	195 low status	STAI (mean scores)	Pregnancy	State anxiety: 40.67 Trait anxiety: 46.40	
Giardinelli et al. (2012) Italy [60]	590	STAI-Y (>40) STAI-T (>40) SCID-I	Pregnancy: (28–32 weeks)	State anxiety: 20.5 Trait anxiety: 25.3 GAD: 1.4	
Ibanez et al. (2012) France [61]	1719	STAI-S (≥37)	Pregnancy (24–28 weeks)	7.9	
Figueiredo and Conde (2011) Portugal [62]	260	STAI-S (≥45)	1st trimester 2nd trimester 3rd trimester	13.1 12.2 18.2	
Coelho et al. (2011) United Kingdom [63]	246 with high scores of anxiety	SCID (DSM-IV)	Pregnancy: 2nd trimester	GAD: 22.7	
Uguz et al. (2010) Turkey [16]	309	SCID-I	Pregnancy	GAD: 3.6	
van Bussel et al. (2009) Belgium [64]	403	HADS-A (mean scores) PRAQ-55 (mean scores)	Pregnancy: 8–15 weeks 20–26 weeks 30–36 weeks	HADS-A	PRAQ
				5.0	135.5
				5.0	147.5
				5.4	148.1
Bödecs et al. (2009) Hungary [65]	307	STAI-T (>52)	Pregnancy	14.6	
Borri et al. (2008) Italy [13]	1066	SCID (DSM-IV)	Pregnancy: (12–15 weeks)	1.9	
Felice et al. (2007) Malta [14]	229	CIS-R	Pregnancy (18.6 weeks)	4.4 (anxiety disorders) GAD: 0.4	
Berle et al. (2005) Norway [66]	680	HADS-A (>8)	Pregnancy	10.4	
Sutter-Dallay et al. (2004) France [67]	497	MINI (DSM-IV)	3rd trimester	24.1 (some anxiety disorder) GAD: 8.5	
Andersoon et al. (2003) Sweden [68]	1556	PRIME-MD	2nd trimester	14.1 (some disorder) 6.6 (anxiety disorders) GAD: 0.3	
Zar et al. (2002) Sweden [69]	453	ADIS-R	Pregnancy (28–32 weeks)	GAD: 0.9	
Dayan et al. (2002) France [70]	634	STAI (mean scores)	Pregnancy	State anxiety: 36.4 Trait anxiety: 38.8	
Canals et al. (2002) Spain [71]	96	STAI (mean scores)	Pregnancy: 1st trimester 3rd trimester	STAI-S	STAI-T
				15.3	16.3
				14.7	16.4

## Data Availability

The data presented in the current study are available from the authors upon request.
